# Chloroplast Genome Provides Insights into Molecular Evolution and Species Relationship of Fleabanes (*Erigeron*: Tribe Astereae, Asteraceae) in the Juan Fernández Islands, Chile

**DOI:** 10.3390/plants13050612

**Published:** 2024-02-23

**Authors:** Seon-Hee Kim, JiYoung Yang, Myong-Suk Cho, Tod F. Stuessy, Daniel J. Crawford, Seung-Chul Kim

**Affiliations:** 1 Department of Botany, Graduate School of Science, Kyoto University, Kyoto 606-8502, Japan; desfilles@naver.com; 2 Research Institute for Dok-do and Ulleung-do Island, Kyungpook National University, Daegu 41566, Republic of Korea; jyyangson@gmail.com; 3 Department of Biological Sciences, Sungkyunkwan University, Suwon 16419, Republic of Korea; marina0426@gmail.com; 4 Department of Evolution, Ecology, and Organismal Biology, The Ohio State University, Columbus, OH 43210, USA; stuessy.1@osu.edu; 5 Department of Ecology and Evolutionary Biology and the Biodiversity Institute, The University of Kansas, Lawrence, KS 66045, USA; dcrawfor@ku.edu

**Keywords:** adaptive radiation, *Erigeron*, insular endemic, molecular evolution, positive selection

## Abstract

*Erigeron* represents the third largest genus on the Juan Fernández Islands, with six endemic species, five of which occur exclusively on the younger Alejandro Selkirk Island with one species on both islands. While its continental sister species is unknown, *Erigeron* on the Juan Fernández Islands appears to be monophyletic and most likely evolved from South American progenitor species. We characterized the complete chloroplast genomes of five *Erigeron* species, including accessions of *E. fernandezia* and one each from Alejandro Selkirk and Robinson Crusoe Islands, with the purposes of elucidating molecular evolution and phylogenetic relationships. We found highly conserved chloroplast genomes in size, gene order and contents, and further identified several mutation hotspot regions. In addition, we found two positively selected chloroplast genes (*ccsA* and *ndhF*) among species in the islands. The complete plastome sequences confirmed the monophyly of *Erigeron* in the islands and corroborated previous phylogenetic relationships among species. New findings in the current study include (1) two major lineages, *E. turricola*–*E. luteoviridis* and *E. fernandezia*–*E. ingae*–*E. rupicola*, (2) the non-monophyly of *E. fernandezia* occurring on the two islands, and (3) the non-monophyly of the alpine species *E. ingae* complex.

## 1. Introduction

The Juan Fernández Archipelago in the Pacific Ocean, located 667 km off the west coast of Chile, is composed of two major islands: Masatierra (or Isla Robinson Crusoe (RC)) and Masafuera (Isla Alejandro Selkirk (AS)) [1]. These two islands, RC and AS, are ca. 4 and 1 million years (my) old, respectively [2], and have estimated areas of 48 and 50 km^2^, respectively. The islands are separated by 181 km in an east–west orientation. Despite their relatively small size and young geological age, the Juan Fernández Islands harbor a flora with 65% percent endemic species for angiosperms) [3,4,5]. A combination of small flora and the simplicity of the biogeographic setting makes this archipelago ideal for biogeographic and evolutionary studies, especially for assessing the patterns of the two modes of speciation (i.e., cladogenesis and anagenesis) and their genetic consequences [6,7].

After the two endemic genera *Dendroseris* D. Don and *Robinsonia* DC., *Erigeron* L. is the third most species-rich genus on Juan Fernández with six endemic species: *E*. *fernandezia* (Colla) Harling, *E*. *ingae* Skottsb., *E*. *luteoviridis* Skottsb., *E*. *rupicola* Phil., *E*. *stuessyi* Valdeb. and *E*. *turricola* Skottsb. [8]. Unlike *Robinsonia* and *Dendroseris* species that primarily occur on the older island (Robinson Crusoe), five of the six *Erigeron* species are restricted to the younger island (Alejandro Selkirk); *E*. *fernandezia* also occurs on Robinson Crusoe [8]. Morphological and limited nucleotide sequence data suggested that the group in the islands has resulted from a single introduction, likely from a single continental ancestor in South America, where the genus is diverse [8,9,10]. All endemic species are polyploid (*n* = 27) [11,12,13,14]. Species differ in leaf shape (lanceolate to spatulate), capitulum size and number, and growth form (semiglobose subshrubs to erect shrubs over 1 m tall) [8]. In addition, ecological differentiation can be observed from near sea level to over 1000 m, from dry and exposed areas near the coast to wet environments usually covered by fog [8]. A cladistic analysis of morphological characters revealed two major clades: one containing two species, *E*. *rupicola*–*E*. *stuessyi*, and the other containing the remaining four species, i.e., the *E*. *ingae* complex. Additionally, a sister relationship between *E*. *luteoviridis* and *E*. *fernandezia* was suggested [8]. Recently, two nuclear markers, amplified fragment length polymorphisms (AFLPs) and simple sequence repeats (SSRs), were explored to determine genetic variation within and differentiation among six species of *Erigeron* [15]. This study found the following three distinct genetic lineages within the genus: (1) *E*. *rupicola* and *E*. *stuessyi*, (2) *E*. *fernandezia*, and (3) the *E*. *ingae* complex (including *E*. *ingae*, *E*. *luteoviridis* and *E*. *turricola*). In the case of *E*. *fernandezia* on two islands, the results are suggestive of its origin on the younger Alejandro Selkirk Island with the subsequent dispersal and establishment on the older Robinson Crusoe Island [15].

The rapid accumulation of the complete plastome sequences has provided valuable information both for resolving phylogenetic relationships at various taxonomic levels and for building reference genomes. As examples, we have gained pivotal information from plastome sequences for inferring the origin and evolution of plant endemics in island settings: *Zanthoxylum* [16], *Rubus* [17], *Fagus* [18], *Dendroseris* [19], *Viola* [20], the woody *Sonchus* alliance [21], etc. In particular, the plastid phylogenomic analysis of *Dendroseris*, the largest endemic genus in the Juan Fernández Islands, supported the monophyly of the genus and the full resolution of interspecific and intersubgeneric relationships [19]. In addition, mutation hotspots and highly variable chloroplast microsatellites (simple sequence repeats (SSRs)) were identified as potential chloroplast markers.

While complete plastome sequences for certain plant groups have recently been generated as genomic references and for phylogenomic investigations at various taxonomic levels [17,19,22,23,24,25], very little is known about the plastomic genomes reference of *Erigeron* and related genera, including *Conyza* [26,27]. In particular, the plastomes of tribe Astereae from the Southern Hemisphere, including subtribe Conyzinae, are poorly represented, and both specific and higher-level relationships in *Erigeron* are poorly understood [10]. Although some insights into species relationships and genetic variation among endemic species of *Erigeron* in the Juan Fernández Islands have been provided in previous studies, no attempt has been made to assemble complete plastomes to generate genomic reference, to understand molecular evolution, nor to further confirm species relationships. Therefore, the purposes of this study are to (1) characterize much needed plastomes of *Erigeron* species to build genomic references for future phylogenomic analysis of the genus on a global scale, (2) conduct a comprehensive comparative plastome analysis among five species of *Erigeron* to understand plastome evolution on oceanic islands and (3) assess the first phylogenetic relationships among species based on complete plastome sequences to gain insights into species relationships.

## 2. Results

### 2.1. Genome Size and Features

**Figure 1:**
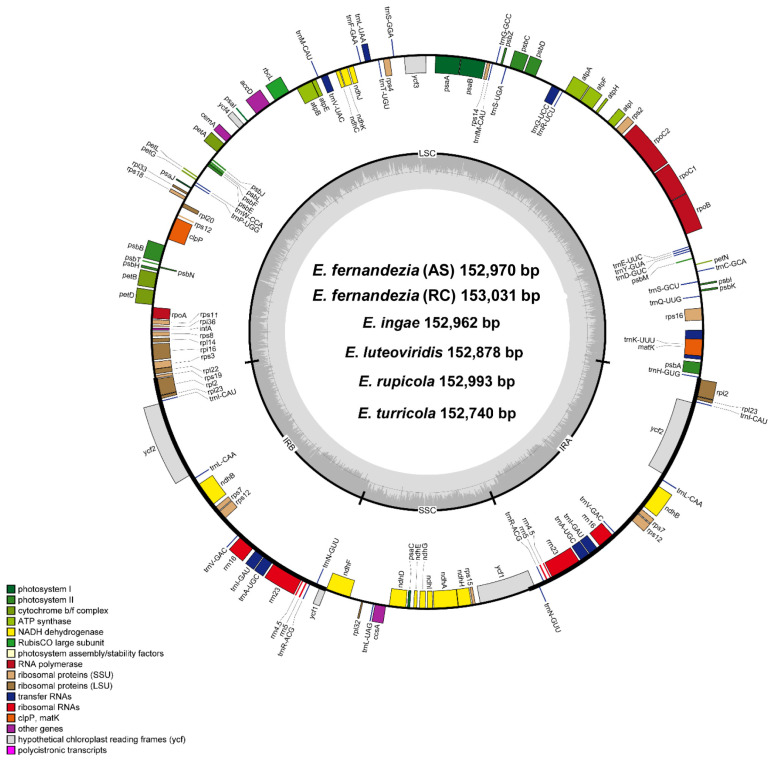
Complete plastome map of six *Erigeron* species. The genes located outside of the circle are transcribed counterclockwise, while those located inside are transcribed clockwise.

With the exception of *E*. *stuessyi*, the six complete plastomes of five *Erigeron* species from the Juan Fernández Islands were characterized for the first time: two accessions of *E*. *fernandezia* in Alejandro Selkirk (AS) and Robinson Crusoe Island (RC), *E*. *ingae*, *E*. *luteoviridis*, *E*. *rupicola* and *E*. *turricola*. The length of complete plastome sequences ranged from 152,740 bp (*E*. *turricola*) to 153,031 bp (*E*. *fernandezia*_RC), and the plastomes were highly conserved with no structural variation or content rearrangements (Figure 1). The large single-copy (LSC) region, small single-copy (SSC) region, and two inverted repeat (IR) regions ranged from 84,864 bp (*E*. *turricola*) to 84,504 bp (*E*. *fernandezia*_RC), from 18,355 bp (*E*. *fernandezia*_RC) to 18,219 bp (*E*. *luteoviridis*), and from 25,027 bp (*E*. *luteoviridis*) to 24,770 bp (*E. turricola*), respectively (Table 1). The plastomes of *Erigeron* species on the islands contained 130 genes, including 85 protein-coding genes, except for *E*. *rupicola* which contained 131 genes (one additional *rps19* pseudogene present). All six newly sequenced plastomes of *Erigeron* contained eight rRNA and 36 tRNA genes, and their overall guanine–cytosine (GC) content was identical, i.e., 37.2%. Also, all six plastomes contained a total of 17 duplicated genes in the IR regions, including seven tRNA, four rRNA and six protein-coding genes. Fifteen genes (*ndhA*, *ndhB*, *petB*, *petD*, *rpl2*, *rpl16*, *rpoC1*, *rps12*, *rps16*, *trnA*-UGC, *trnG*-UCC, *trnI*-GAU, *trnK*-UUU, *trnL*-UAA and *trnV*-UAC) contained a single intron, whereas *clpP* and *ycf3* each contained two introns.

For the pseudogenized *ycf1* gene, which is positioned in the IRb/SSC junction region, *E*. *luteoviridis* contained the longest one (732 bp), while the others contained the shorter and same size of 597 bp. As for the complete *ycf1* gene in the SSC/IRa junction, *E*. *rupicola* and *E. turricola* contained the short gene (4524 bp and 4470 bp, respectively), whereas the others all had the same length of 5055 bp. Interestingly, five plastomes of *Erigeron* contained one single complete *rps19* gene (279 bp), whereas *E*. *rupicola* retained two *rps19* genes, including one pseudogenized gene (123 bp) and one single complete gene (279 bp).

**Figure 2:**
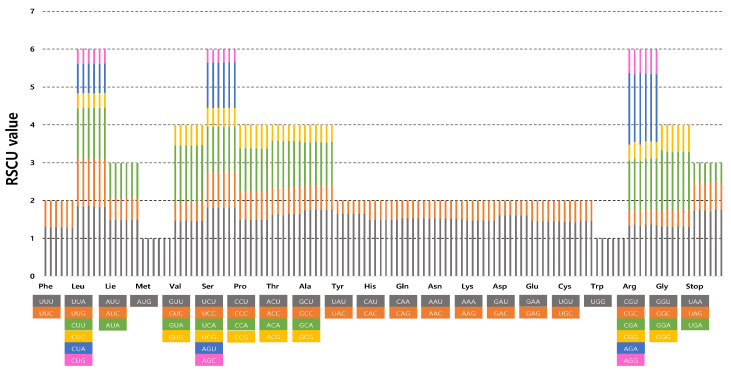
Relative synonymous codon usage in six newly sequenced *Erigeron* accessions. The list of species from left to right columns represent *E*. *fernandezia* (AS), *E*. *fernandezia* (RC), *E*. *ingae*, *E*. *luteoviridis* and *E*. *turricola*.

The frequency of codon usage of the six complete plastomes of *Erigeron* was calculated based on the sequences of protein-coding and tRNA genes. The results showed that the average codon usage among the six accessions ranged from 23,892 (*E*. *turricola*) to 25,766 (*E*. *ingae*) (Supplementary Table S1). The average codon usage for the remaining species was 25,656 for *E*. *fernandezia* (AS), 24,024 for *E*. *fernandezia* (RC), 25,766 for *E*. *luteoviridis*, 24,002 for *E*. *rupicola* and 23,892 for *E*. *turricola*. The highest RSCU value was indicated in the usage of the UUA codon for leucine (1.82–1.86) followed by GCU for alanine (1.75–1.76) and AGA for arginine (1.79–1.89). The lowest RSCU value was indicated in the usage of AGC for serine (0.35–0.36) and GAC for aspartic acid (0.39–0.40). We found the distribution of codon types to be consistent (Figure 2), and codons AUG (M) and UGG (W) encoded methionine and tryptophan, respectively, showing no bias (RSCU = 1) (Supplementary Table S1).

### 2.2. Comparative Analysis of Genome Structure

**Figure 3:**
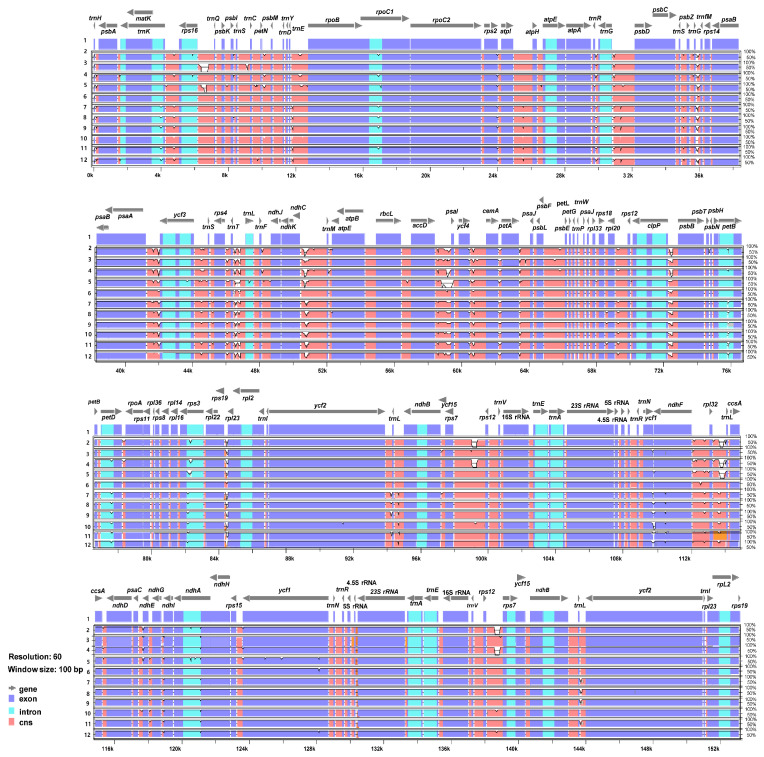
Visualization of alignment of the 12 plastome sequences of *Erigeron* accessions. Species: 1. *E. annuus* MZ361990; 2. *E. breviscapus* NC043882; 3. *E. canadensis* NC046789; 4. *E. multiradiatus* NC056169; 5. *E. philadelphicus* MT579973; 6. *E. strigosus* MT579973; 7. *E. fernandezia* (AS); 8. *E. fernandezia* (RC); 9. *E. ingae*; 10. *E. luteoviridis*; 11. *E. rupicola*; 12. *E. turricola*.

**Figure 4:**
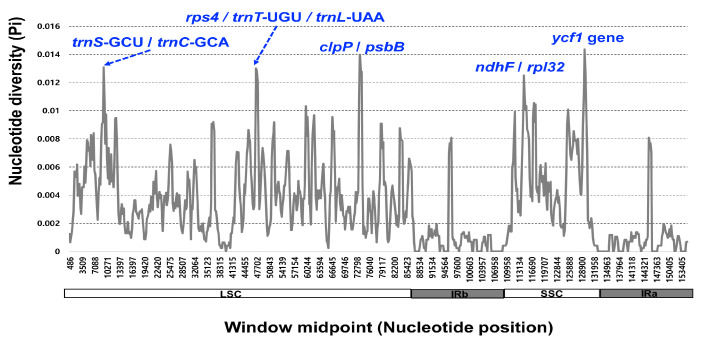
Sliding window analysis of the 12 whole chloroplast genomes of *Erigeron* species.

The 11 complete plastome sequences of the *Erigeron* accessions were plotted using mVISTA analysis, using the annotated *E*. *annuus* plastome as a reference (Figure 3). The results indicated that the LSC region was the most divergent, that the two IR regions were highly conserved, and that the non-coding regions were more divergent and variable than the coding regions. A sliding window analysis using the DnaSP program revealed highly variable regions in 11 *Erigeron* chloroplast genomes (Figure 4). The average value of nucleotide diversity (PI) over the entire cp genome was 0.0033. The most variable region was *ycf1* genic region with a PI value of 0.01436. Also, highly variable regions included four other intergenic regions, i.e., *clpP*/*psbB* (Pi = 0.01398), *rps4*/*trnT*-UGU/*trnL*-UAA (Pi = 0.013), *trnS*-GCU/*trnC*-GCA (Pi = 0.01309) and *ndhF*/*rpl32* (Pi = 0.0125). Therefore, five highly variable regions with PI value of greater than 0.012 were identified in 11 *Erigeron* plastid genomes, which can be used for population genetic and phylogeographic studies. A positive selection analysis allowed us to identify two positively selected genes, with a statistically significant LRT *p* value based on the M7 and M8 model, among the six *Erigeron* plastomes on the Juan Fernández Islands (Table 2); they are the *ccsA* and *ndhF* genes, subunits of the cytochrome and NADH-dehydrogenase subunit genes, respectively. However, all but two genes, i.e., 73 of the 75 genes, had an average Ka/Ks ratio below 1, indicating that these genes have been subjected to strong purifying selection in the insular *Erigeron* chloroplast genomes.

### 2.3. Phylogenetic Analysis

**Figure 5:**
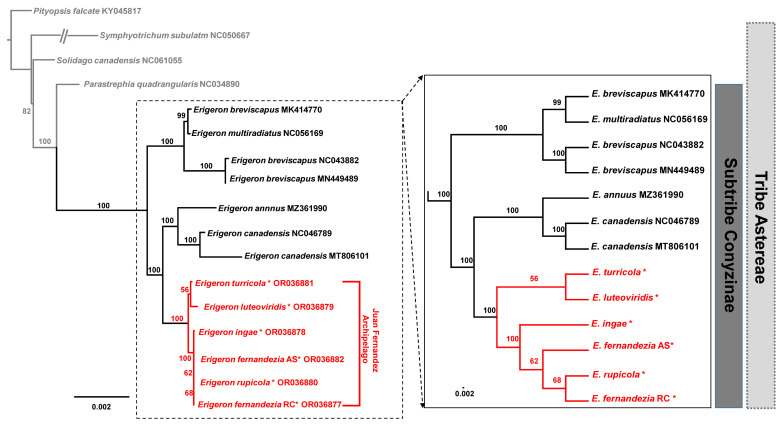
The maximum-likelihood (ML) tree inferred from 15 accessions of *Erigeron* and six Astereae tribe plastomes. The bootstrap value based on 1000 replicates is shown on each node. The six newly sequenced accessions in the current study are indicated using red asterisks.

A maximum likelihood (ML) analysis based on the concatenated 80 common protein-coding genes, excluding the ones duplicated in the IR regions, was conducted on the best-fit model of TPM3u+F+I. A total of 62,990 aligned nucleotide bases included 685 parsimony-informative sites. A phylogenetic analysis of 21 representative plastomes within the Astereae tribe confirmed monophyly of the *Erigeron* genus (100% bootstrap support (BS)) from the outgroup taxa (*Parastrephia quadrangularis*, *Pityopsis aspera* var. *adenolepis*, *P*. *falcata*, *P*. *graminifolia*, *Symphyotrichum subulatum* and *Solidago canadensis*) (Figure 5). Within *Erigeron*, two Old World species, *E. breviscapus* and *E*. *multiradiatus*, formed an early divergent lineage, but *E*. *breviscapus* appeared to be not monophyletic (100% BS). The remaining species of *Erigeron* formed two major clades, one including continental herbaceous species (*E*. *canadensis*, *E*. *philadelphicus*, *E*. *annuus* and *E*. *strigosus*; 100% BS) and the other including Juan Fernández Islands endemics (*E*. *turricola*, *E*. *luteoviridis*, *E*. *fernandezia*, *E*. *ingae* and *E*. *rupicola*; 100% BS) (Figure 5). Within the monophyletic *Erigeron* on the Juan Fernández Islands, two major lineages could be further recognized: one includes *E*. *turricola*-*E*. *luteoviridis* (56%) and the other includes *E*. *fernandezia*-*E*. *ingae*-*E*. *rupicola* (100% BS). The only species found on both Alejandro Selkirk (AS) and Robinson Crusoe Island (RC), *E*. *fernandezia*, turned out to be not monophyletic; *E*. *fernandezia* on RC is sister to *E*. *rupicola* (68% BS).

## 3. Discussion

### 3.1. Chloroplast Genome Structure and Evolution in Genus Erigeron

In this study, we newly sequenced and characterized five species of *Erigeron* (six accessions): *E*. *fernandezia* (AS), *E*. *fernandezia* (RC), *E*. *ingae*, *E*. *luteoviridis*, *E*. *rupicola* and *E*. *turricola*. As the cp genomes are generally conserved in sequence and structure in land plants [28], we found highly conserved cp genomes in the genus *Erigeron*, especially among the species on the Juan Fernández Islands (Table 1). The high conservation of plastomes in *Erigeron* is consistent with the largest endemic genus *Dendroseris* on the Juan Fernández Islands, which radiated on the older Robinson Crusoe Island [19].

While the chloroplast genomes were highly conserved amongst the 11 *Erigeron* species (six continental and five Juan Fernández Islands), we identified five divergence hotspot regions (*ycf1*, *clpP*/*psbB*, *rps4*/*trnT*-UGU/*trnL*-UAA, *trnS*-GCU/*trnC*-GCA and *ndhF*/*rpl32*) based on mVISTA and sliding window analysis (Figure 3 and Figure 4). With the exception of two regions, these regions are different from the eight hotspot regions based on the 11 continental *Erigeron* species only: *accD*/*psaI*, *trnT*, *ndhC*/*trnV*, *clpP*/*psbB*, *trnT*/*trnL*, *trnG*/*trnfM*, *psaA*/*ycf3* and *ccsA*/*rpl32* [29]. In comparison to the five mutation hotspot regions of insular *Erigeron* (the Astereae tribe) with those in the genus *Dendroseris* (the Cichorieae tribe), two regions, *ycf1* and *trnT*/*trnL*, are found to be highly variable [19]. Highly variable chloroplast regions have been widely utilized for species delimitation, phylogenetic inference, and infraspecific phylogeography [30,31,32,33,34,35,36]. Therefore, we suggest that several variable chloroplast regions found in *Erigeron* may serve as effective DNA barcoding markers and could be useful for *Erigeron* phylogenetic and infraspecific phylogeographic studies.

We also found that two chloroplast genes, *ccsA* and *ndhF*, were under positive selection, cautiously indicating their adaptive roles during the diversification of *Erigeron* on the Juan Fernández Islands. The *ccsA* (*ycf5*) gene encodes a protein required for cytochrome biogenesis that mediates the attachment of heme to c-type cytochromes [37]. It has been shown that the *ccsA* gene was under positive selection for epiphytic orchid and fig species as well as insular endemic *Cotoneaster wilsonii* [38,39,40]. Although *ccsA* may play an important role in the adaptation of epiphytes to special habitats, it is unclear how this gene is under positive selection for *Erigeron* species on the Juan Fernández Islands. It is plausible that positive selection on *ccsA* may contribute to adaptation to specific environmental conditions, such as light intensity, moisture, and/or temperature on oceanic islands [39]. The other selected *ndhF* gene encodes NADH dehydrogenase subunit proteins and has been known to be under positive selection in various plant groups, including the ones adapted to different altitudinal habitats and in shade-tolerant and sun-loving plants [17,18,41,42,43,44,45,46]. The adaptive evolution of the *ndhF* gene may affect energy transformation and resistance to photo-oxidative stress in different environments [47]. Furthermore, the *ndhF* gene is known to be involved in adaptation to hot and dry climates, likely contributing to adaptation to high light intensity during the evolution of *Erigeron* on the Juan Fernández Islands [46,48,49].

### 3.2. Adaptive Radiation of Erigeron on the Younger Island of the Juan Fernández Archipelago

*Erigeron* is biogeographically and evolutionarily interesting because its colonizing ancestor apparently became established on the younger island Alejandro Selkirk Island and later dispersed to the older island Robinson Crusoe. Upon colonization on the younger island, cladogenetic speciation resulted in the complex of six species within 1 million years [15]. Given the ecological differentiation among species, *Erigeron* represents the best example of radiation, presumably adaptive, within the archipelago: *E*. *rupicola* (coastal rocks), *E*. *stuessyi* (lower elevations, but only inside the deep, cool, and moist ravine walls), *E*. *fernandezia* (open areas, especially on the middle slopes and ravine margins), and *E*. *ingae*/*E*. *luteoviridis*/*E*. *turricola* (higher elevation, ca. 800–1200 m, in the fern–grassland mosaic zone). Morphologically, *E*. *rupicola* is a small rosette herb with short flowering stalks and small solitary heads, and the closely related *E*. *stuessyi* can be distinguished from it by having thinner leaves with longer flowering stalks [15]. *Erigeron fernandezia* is tall, with long, dentate leaves (sometimes woody at the base) and with many medium-sized heads. The remaining three species of the *E*. *ingae* complex are morphologically similar, having rosette-bearing and long flowering stalks with many larger flowering heads.

While our overall phylogenetic relationships among species and their biogeographic scenarios tend to be consistent with the previous AFLP and microsatellite data [15], one unexpected finding based on complete plastome data is the position of *E*. *ingae* (Figure 5). Rather than forming a clade with two other morphologically similar, higher-elevation species (ca. 800–1200 m), i.e., *E*. *turricola* and *E*. *luteoviridis*, *E*. *ingae* is closely related to lower/middle-elevation species, *E*. *rupicola* (50–100 m) and *E*. *fernandezia* (below 100 m to ca. 1200 m; AS). Given its unusual position based on complete plastome, it is possible that higher-elevation *E*. *ingae* experienced gene flow with either strictly coastal *E*. *rupicola* and lower/middle-elevation *E*. *fernandezia* or their most recent common ancestor. An unrooted neighbor-joining tree based on microsatellites (Figure 3 of López-Sepúlveda et al. [15]) showed that of the two allopatric populations of *E*. *ingae* sampled, one population (population 33) is clustered with the geographically closer *E*. *fernandezia* (population 16; AS) and *E*. *luteoviridis* (population 36), while the other one is clustered with *E*. *turricola* (populations 46 and 47) and *E*. *fernandezia* (population 22; AS). Considering the geographical distribution of these species in the western part of AS, *E*. *ingae* likely captured the chloroplast from *E*. *fernandezia* (AS) rather than from the primarily eastern coastal species *E*. *rupicola*. Alternatively, incomplete lineage sorting when undergoing rapid adaptive radiation might likely be the cause of this incongruence, which requires further investigation based on more genome-wide, unlinked markers [50,51].

Both the AFLP and microsatellite data clearly demonstrated the population differentiation of *E*. *fernandezia* on the two islands and showed lower diversity in *E*. *fernandezia* on RC than on AS, suggesting its origin on the younger AS with subsequent dispersal to the older RC [15]. Morphologically the populations of *E*. *fernandezia* do not differ between the two islands, and given their restricted distribution to paths and disturbed areas, the older island RC populations were suggested to have been introduced back even during historical time [8,52]. Differences in the flavonoid profiles further support population differentiation between the two islands; the majority of RC populations contain C-glycosyl-flavones, whereas AS populations lack them [8]. Our current study also finds chloroplast genome divergence between the two islands and thus infraspecific population divergence between the two islands, certainly more than among species of the *E*. *ingae* complex [15]. In addition, no significant genetic reduction in RC populations and population differentiation patterns were demonstrated by López-Sepúlveda [15], likely refuting the possibility of the recent, especially during historical time, origin of *E*. *fernandezia* on RC.

The three species of *E*. *ingae* complex appear to be segregated from other taxa morphologically and genetically, despite not being ecologically partitioned. The taxonomic treatment of *E*. *turricola* as a heterotypic synonym of *E*. *ingae* [53] was not supported by the previous AFLP/microsatellite study. *Erigeron turricola* was genetically diverged from *E*. *ingae* based on microsatellites (F_ST_ = 0.422 vs. F_ST_ = 0.269 between *E*. *luteoviridis* and *E*. *fernandezia*, AS; and F_ST_ = 0.231 between *E*. *turricola* and *E*. *fernandezia*, AS), while the AFLP data showed the lowest genetic differentiation between the two species (F_ST_ = 0.095). The current study suggested the sharing of their most recent common ancestor between *E*. *turricola* and *E*. *luteoviridis*. It is uncertain whether the three alpine species of the *E*. *ingae* complex are monophyletic, and the monophyly of each species and their phylogenetic relationships on AS await determination. Given the non-monophyly of the three *E*. *ingae* complex species based on the current plastome relationships and morphological similarity between *E*. *fernandezia* and *E*. *luteoviridis* (even once considered conspecific with *E*. *turricola* [53]), it is plausible that *E*. *ingae* and *E*. *luteoviridis* independently adapted to the alpine conditions and drier subalpine regions, respectively. Owing to the potential difficulty in interpreting banding patterns of AFLPs and microsatellites of hexaploid (*n* = 27) species such as *Erigeron* as contrasted interpreting bands in diploid plants, the methods used for inferring phylogenetic relationships of diploids are not adequate for polyploids. The use of new analyses for polyploids [54,55,56] may provide insights into the evolutionary history and adaptive radiation of *Erigeron* in the Juan Fernández Islands.

In conclusions, we characterized the complete chloroplast genomes of five *Erigeron* species, building important genomic references for the future global-scale phylogenomic analysis of the genus. Chloroplast genomes were found to be highly conserved and provided novel phylogenetic relationships among species in the Juan Fernández Islands. We identified five divergence hotspot regions of insular *Erigeron*, which might be useful as effective DNA barcoding markers and in infraspecific phylogeographic studies. Two chloroplast genes, *ccsA* and *ndhF*, were positively selected, likely contributing to the adaptation and speciation of *Erigeron* in the Juan Fernández Islands.

## 4. Materials and Methods

### 4.1. Plant Sampling, DNA Isolation, and Plastome Sequencing, Assembly and Annotation

Given the rarity and conservation status of some *Erigeron* species on the islands, we selected and sequenced six complete plastome of *Erigeron* accessions in this study: two accessions of *E*. *fernandezia*, one on Alejandro Selkirk (AS) and the other on Robinson Crusoe Island (RC), and one accession each of *E*. *ingae*, *E*. *luteoviridis*, *E*. *rupicola*, and *E*. *turricola*. We were not able to include *E*. *stuessyi* due to sequencing difficulty. Silica gel dried leaf tissues collected in the field during the previous expeditions or herbarium specimens deposited at OS (the Ohio State University herbarium) were used for DNA extraction (Supplementary Table S2). Total DNA was extracted using the DNeasy Plant Mini Kit (Qiagen, Carlsbad, CA, USA) and sequenced using an Illumina HiSeq 4000 (Illumina, Inc., San Diego, CA, USA), yielding a 150 bp paired-end read length, at Macrogen Co. (Seoul, Republic of Korea). The resulting paired-end reads were assembled de novo using Velvet v1.2.10 with multiple k-mers [57] with coverage ranging from 71 to 291. tRNAs were confirmed using tRNAscan-SE [58]. The sequences were annotated using Geneious R10 [59] and deposited in GenBank (OR036877-OR036882). Annotated sequence files in the GenBank format were used to draw a circular map with OGDRAW v1.2 [60].

### 4.2. Comparative Plastome Analysis

To understand plastome evolution within the genus *Erigeron*, we selected 11 taxa (12 accessions) including six newly assembled plastomes of *Erigeron* [six continental species, i.e., *E*. *annus* (MZ361990), *E*. *breviscapus* (NC043882), *E*. *canadensis* (NC046789), *E*. *multiradiatus* (NC056169), *E*. *philadelphicus* (MT579972), *E*. *strigosus* (MT579973), and five insular endemics, i.e., *E*. *fernandezia* (two accessions, one each from AS and RC), *E*. *ingae*, *E*. *luteoviridis*, *E*. *rupicola*, and *E*. *turricola*)] and compared their genomic features using the Shuffle-LAGAN mode [61] of mVISTA [62]. The sequences of the 12 *Erigeron* plastomes were aligned using the back-translation approach with MAFFT ver.7 [63] and were manually edited using Geneious R10 [59]. Using DnaSP 6.10 [64], a sliding window analysis was performed with a step size of 200 bp and window length of 800 bp to determine the nucleotide diversity (Pi) of the plastomes. The codon usage frequency was calculated using MEGA 7 [65] based on the relative synonymous codon usage (RSCU) value [66], which is a simple measure of the non-uniform usage of synonymous codons in a coding sequence. The DNA code used by bacteria, archaea, prokaryotic viruses and chloroplast proteins was used [67]. To evaluate the natural selection pressure on the protein-coding genes of six *Erigeron* plastomes, a site-specific model was tested using EasyCodeML [68] with the CODEML algorithm [69]. Seven codon substitution models (M0, M1a, M2a, M3, M7, M8 and M8a) were compared to detect the positively selected sites based on the likelihood ratio test (LRT).

### 4.3. Phylogenetic Analysis

For the phylogenetic analysis, complete plastome sequences of 15 accessions of *Erigeron* and six representative species of outgroup within the Asteraceae tribe were aligned using MAFFT ver.7 [63] in Geneious R10 [59]. The 15 accessions of the ingroup *Erigeron* included all nine *Erigeron* plastome sequences currently available in GenBank with two additional accessions of *E*. *breviscapus* (MK414770 and MN449489) and *E*. *canadensis* (MT806101) and the six newly assembled *Erigeron* plastome sequences in this study. For the concatenated sequences of 80 common protein-coding genes (excluding the ones duplicated in the IR regions), a maximum likelihood (ML) analysis based on the best-fit model of “TPM3u+F+I” was conducted using IQ-TREE 1.4.2 [70]. *Parastrephia quadrangularis* (NC034890), *Solidago canadensis* (NC061055), *Symphyotrichum subulatum* (NC050667) and three plastomes of *Pityopsis* (*P*. *aspera* var. *adenolepis* (MW271041), *P*. *falcata* (KY045817) and *P*. *graminifolia* (MW279328)) were used as the outgroup, and a non-parametric bootstrap analysis was performed with 1000 replicates.

## Figures and Tables

**Table 1:** Summary of the characteristics of the six *Erigeron* chloroplast genomes on the Juan Fernández Islands.

Taxa	*E. fernandezia* (AS)	*E. fernandezia* (RC)	*E. ingae*	*E. luteoviridis*	*E. rupicola*	*E. turricola*
Total cpDNA size (bp)	152,970	153,031	152,962	152,878	152,993	152,740
GC content (%)	37.2%	37.2%	37.2%	37.2%	37.2%	37.2%
LSC size (bp)/GC content (%)	84,611/35.1%	84,630/35.1%	84,597/35.1%	84,605/35.1%	84,635/35.1%	84,864/35.1%
IR size (bp)/GC content (%)	25,012/43.0%	25,023/43.0%	25,012/43.0%	25,027/43.0%	25,012/43.0%	24,770/43.0%
SSC size (bp)/GC content (%)	18,335/31.0%	18,355/31.0%	18,341/31.0%	18,219/30.9%	18,334/31.0%	18,336/30.9%
Number of genes	130	130	130	130	131 *	130
Number of protein-coding genes	85	85	85	85	85	85
Number of tRNA genes	36	36	36	36	36	36
Number of rRNA genes	8	8	8	8	8	8
Number of duplicated genes	17	17	17	17	17	17
Accession Number	OR036882	OR036877	OR036878	OR036879	OR036880	OR036881

AS: Alejandro Selkirk Island; RC: Robinson Crusoe Island. LSC: large single copy region, IR: inverted repeat, SSC: small single copy region. *: includes one additional *rps19* pseudo gene.

**Table 2:** Log-likelihood values of the site-specific models based on six accessions of *Erigeron* on the Juan Fernández Islands, with detected sites having dN/dS values > 1.

Gene Name	Models	np	ln L	Likelihood Ratio Test *p*-Value	Positively Selected Sites
*ccsA*	M8	14	−1274.227378	0.000000000	94 Q 0.967 *
	M7	12	−1295.780345		
*ndhF*	M8	14	−2751.256419	0.000000315	702 P 0.996 **
	M7	12	−2766.227043		

* *p* < 0.05; ** *p* < 0.01; np represents the number of parameters for corresponding model.

## Data Availability

The data presented in this study are openly available in the NCBI GenBank (https://www.ncbi.nlm.nih.gov/) (accession numbers: OR036877-OR036882O).

## Supplementary Materials

The following supporting information can be downloaded at: https://www.mdpi.com/article/10.3390/plants13050612/s1, Table S1: The codon usage and codon–anticodon recognition pattern for tRNA in the six *Erigeron* plastomes from the Juan Fernández Islands. Table S2: List of six *Erigeron* accessions obtained from the Juan Fernández Islands. NA = not available. AS = Alejandro Selkirk Island. RC = Robinson Crusoe Island.
